# Isolated Metachronous Splenic Metastasis of Lung Adenocarcinoma: Long‐Term Survival After Splenectomy

**DOI:** 10.1155/crom/1635762

**Published:** 2026-04-17

**Authors:** Nina Wittlin, Martin Buess

**Affiliations:** ^1^ Faculty of Medicine, Basel University, Basel, Switzerland, unibas.ch; ^2^ Department of Medical Oncology, St. Claraspital, Basel, Switzerland, claraspital.ch

**Keywords:** case report, lung cancer, splenectomy, splenic metastasis

## Abstract

Metastasis to the spleen is an uncommon finding in lung cancer and is typically associated with multivisceral disseminated disease. Isolated splenic metastasis in patients with lung cancer is exceedingly rare, with only 50 cases reported in the literature. We report a case of an asymptomatic, metachronous, isolated splenic metastasis in a 66‐year‐old female patient diagnosed 18 months after resection of lung adenocarcinoma. The patient underwent diagnostic and therapeutic splenectomy and remained tumor‐free for 14 years. To our knowledge, this represents the longest reported survival following an isolated splenic metastasis from lung cancer. This case suggests that in well‐selected patients with isolated splenic involvement, aggressive local therapy such as splenectomy can be curative and may lead to exceptional long‐term survival.

## 1. Introduction

Lung cancer remains a leading cause of cancer‐related mortality worldwide [1], with the prognosis largely depending on the disease stage. Lung carcinomas are mostly diagnosed at metastatic Stage IV [2]. As found in autopsy and population‐based studies, the most common metastatic sites include the respiratory system, liver, bone, nervous system, and adrenal gland [3–5]. While metastasis to these common sites is well documented, metastasis to the spleen is considered uncommon, with isolated and metachronous splenic metastases being extremely rare [6].

Evidence for isolated splenic involvement in lung cancer patients is limited to 50 case reports in the literature, as splenic involvement typically occurs in the context of multivisceral disseminated disease. The rarity of clinically detectable splenic metastasis is thought to be associated with the inhibitory mechanisms of the splenic microenvironment, although the underlying pathophysiology is not fully understood [7].

We herein report a case of a patient with lung adenocarcinoma who developed an isolated metachronous splenic metastasis 18 months after the initial diagnosis. This case is notable not only due to the rarity of isolated splenic metastasis in lung cancer but also for the patient’s remarkable 14‐year survival following surgical intervention, surpassing all reported survival times in previously published case studies. Furthermore, we provide a comprehensive review of 49 case reports from both English and non‐English literature to highlight diagnostic and therapeutic aspects.

## 2. Case Report

A 66‐year‐old female with a history of chronic obstructive pulmonary disease (COPD) and chronic coronary artery disease was diagnosed in July 2008 with an adenocarcinoma of the lung at St. Claraspital, Basel, Switzerland. She is a former smoker (50 py), having quit 2 years ago. Written informed consent for the publication of the medical case details was obtained from the patient.

The patient initially presented with night sweats and generalized pruritus during a routine medical checkup with her general practitioner. The suspicion of lung cancer arose when a pulmonary nodule was detected on a chest X‐ray. Subsequent CT imaging further suggested a central bronchial carcinoma of the right lung.

Bronchoscopy revealed a poorly differentiated adenocarcinoma of the lung. Immunohistochemistry was strongly positive for TTF‐1 and negative for CK5/6, CK7, and p63. Molecular characterization was not performed as standard practice at that time. FDG‐PET imaging showed increased uptake in the primary tumor of the right upper lobe, accompanied by a dorsally located satellite lesion, right hilar lymph nodes, and a high suspicion of precarinal lymph node invasion on the right side, without further distant metastases (cTXcN2cM0). Brain metastases were excluded by MRI of the neurocranium. The staging mediastinoscopy histologically confirmed N2 mediastinal lymph node involvement. Pulmonary and cardiac function testing allowed resection up to a right pneumonectomy.

The patient first underwent three cycles of neoadjuvant chemotherapy with cisplatin 100 mg/m^2^ d1 and docetaxel 85 mg/m^2^ d1 q21 as part of the *SAKK16/00* trial [8]. A restaging PET/CT scan demonstrated a significant metabolic response to chemotherapy, with decreased metabolic activity in both the primary tumor and mediastinal lymph nodes. Morphologically, the size of the primary tumor remained unchanged; however, the mediastinal lymph nodes showed a reduction in size.

The patient underwent a right upper lobectomy with radical mediastinal lymph node dissection. Histopathology reported two tumor foci within the right upper lobe with a vital tumor cell content of over 90%. The maximum tumor diameter was 27 mm, and resection margins were tumor‐free (R0). Seven out of 14 dissected regional lymph nodes showed metastatic infiltration, while resection margins remained tumor‐free (R0). Additionally, a hamartoma was identified in the right lower lobe with no evidence of malignancy. Consequently, the pathological TNM stage was determined as *ypT4, ypN2 (7/14), L1, V0,* Stage IIIa according to the 6th UICC classification.

**Figure 1:**
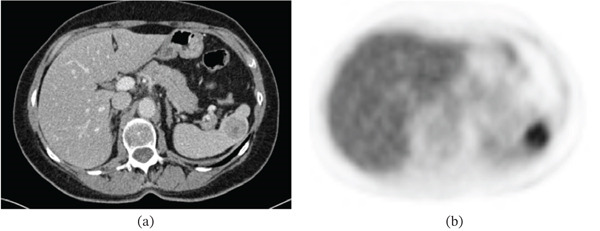
(a) CT and (b) PET images of the abdomen revealing a solitary splenic lesion. The lesion is hypodense on CT and hypermetabolic on PET. It was later confirmed as an isolated splenic metastasis from lung adenocarcinoma.

Eighteen months later, in January 2010, during prescheduled follow‐up within the *SAKK16/00* trial, CT imaging revealed a new splenic lesion (Figure 1)a. A subsequent PET scan found a hypermetabolic mass in the spleen measuring 3 × 3.5 × 3 cm with an SUVmax of 4.6. These findings were highly suggestive of an isolated metachronous splenic metastasis from the primary lung adenocarcinoma, with no evidence of other distant spread or a second primary tumor (Figure 1)b. Given these findings, we decided to perform a diagnostic and therapeutic splenectomy. Preoperatively, the patient was immunized against *Neisseria meningitidis* and *Haemophilus influenzae*. In February 2010, a laparoscopic splenectomy was performed. Histopathological evaluation confirmed a 3.2‐cm metastasis of a moderately differentiated papillary adenocarcinoma consistent with the lung primary. The postoperative course was uneventful.

The patient underwent regular follow‐up CT imaging from 2010 to 2014, all of which showed no signs of recurrence or metastasis. Four years postsplenectomy, in 2014, when no prognostic benefit from further imaging was anticipated, the decision was made to discontinue the routine follow‐up CT scans. In December 2023, during an evaluation for an acute infection, an abdominal CT was performed to identify a potential infectious focus; again, no evidence of malignancy or recurrence was found. Consequently, the patient remained cancer‐free until her death in January 2024 at the age of 81, 14 years after splenectomy. Her death was most likely attributable to infection and left‐sided heart failure, unrelated to her history of lung cancer.

## 3. Discussion

This literature review summarizes 50 reported cases of isolated splenic metastases from primary lung cancer published between 1987 and 2025, including the case presented in this report. The search was primarily conducted in PubMed (National Library of Medicine).

Initially, an exploratory search was performed to identify relevant cases and refine search terminology. Subsequently, a systematic search strategy was developed using MeSH terms (“Splenic Neoplasms” [MeSH] OR “splenic metastasis” [tiab] OR “splenic metastases” [tiab] OR “spleen metastasis” [tiab] OR “spleen metastases” [tiab]) AND (“Lung Neoplasms” [MeSH] OR “lung cancer” [tiab] OR “lung carcinoma” [tiab]). In the first phase, the search was restricted to publications indexed as “case reports.” To ensure comprehensiveness, this filter was removed in a second phase to capture relevant cases not formally classified as such.

A complementary search was also performed using Google Scholar. Inclusion criteria were limited to reports of solitary splenic metastases originating from a primary lung neoplasm. Cases involving multiple primary tumors or suspicion of additional metastases were excluded. The selected cases are compiled in Table 1.

**Table 1:** Overview of 50 case reports describing isolated splenic metastasis in lung cancer patients [9–57].

First author (year)	Histological subtype	Timing of metastasis	Sex	Age	Metastasis symptoms	Treatment of the primary tumor	Treatment of the splenic metastasis	Follow‐up
Klein (1987)[10]	Bronchoalveolar carcinoma	20 months	F	57	Abdominal pain	Right lower and middle lobectomy, adjuvant radiotherapy	Splenectomy, adjuvant radiotherapy	Died 49 months after splenectomy
Edelman (1990)[39]	Adenocarcinoma	0 months	F	63	Asymptomatic	Resection of the primary lung tumor	n.a.	n.a.
Scintu (1991)[11]	Large cell anaplastic carcinoma	0 months	M	n.a.	n.a.	Lobectomy	Splenectomy	Alive 41 months after splenectomy
Johansen (1992)[40]	Adenocarcinoma	n.a.	F	55	n.a.	Resection of the primary lung tumor	n.a.	n.a.
Macheers (1992)[12]	Large cell carcinoma	0 months	F	69	n.a.	n.a.	Splenectomy	Died 1 month after splenectomy
Gupta (1993)[13]	Squamous cell carcinoma	0 months	M	58	Splenic rupture, abdominal pain	n.a.	Emergency splenectomy	Died 10 weeks after splenectomy
Kinoshita (1995)[14]	Squamous cell carcinoma	14 months	M	76	Asymptomatic	Left upper lobectomy	Splenectomy	Died 27 months after splenectomy
Song (1998)[41]	n.a.	25 months	M	54	n.a.	Resection of the primary lung tumor	n.a.	n.a.
Takada (1998)[15]	Bronchopulmonary carcinoid tumor	8 years	M	49	Abdominal pain	Right upper lobectomy	Splenectomy	No evidence of recurrence 8 years after splenectomy
Massarweh (2001)[42]	Adenocarcinoma	0 months	M	68	Splenic rupture	n.a.	Emergency splenectomy, adjuvant chemotherapy	n.a.
Yanada (2001)[43]	Adenocarcinoma	20 months	M	58	Elevated tumor marker	Right upper lobectomy	Splenectomy	n.a.
Tomaszewski (2003)[44]	n.a.	0 months	M	68	Asymptomatic	Left upper lobectomy	Splenectomy	n.a.
Schmidt (2004)[16]	Adenocarcinoma	25 months	M	72	Asymptomatic	Left lower lobectomy, adjuvant radiochemotherapy	Splenectomy	No evidence of recurrence 2 years after splenectomy
Lachachi (2004)[17]	Poorly differentiated carcinoma	0 months	M	77	Splenic rupture	Chemotherapy	Emergency splenectomy	Died 4 months after splenectomy
Pramesh (2004)[45]	Squamous cell carcinoma	n.a.	M	55	Asymptomatic	Neoadjuvant chemotherapy, palliative radiotherapy	Best supportive care	n.a.
Yen (2005)[46]	Adenocarcinoma	2 years	M	56	Elevated tumor marker	Left pneumonectomy, adjuvant radiochemotherapy	n.a.	n.a.
Şanlı (2005)[47]	Adenocarcinoma	0 months	M	64	Asymptomatic	Left pneumonectomy, adjuvant chemotherapy	Splenectomy	n.a.
Sánchez‐Romero (2006)[48]	Adenocarcinoma	0 months	M	73	Abdominal pain, constitutional syndrome	Left upper lobectomy	Splenectomy	n.a.
Assouline (2006)[18]	Large cell bronchial carcinoma	21 months	M	77	Abdominal pain, deterioration in general health	Left pneumonectomy	Splenectomy	No evidence of recurrence 2 years after splenectomy
Van Hul (2008)[49]	Adenocarcinoma	2 years	M	67	Asymptomatic	Left pneumonectomy, adjuvant radiotherapy	Splenectomy, adjuvant chemotherapy	No signs of progression after four sessions of chemotherapy
Fujii (2008)[50]	Pleomorphic carcinoma	3 months	M	58	Asymptomatic	Left upper lobectomy	Splenectomy	n.a.
Ando (2009)[57]	Squamous cell carcinoma	10 months	M	71	Asymptomatic	Radiochemotherapy	Splenectomy	n.a.
Chloros (2009)[19]	Squamous cell carcinoma	0 months	M	59	Asymptomatic	Right pneumonectomy	Splenectomy	The postoperative course was uneventful
Tang (2010)[20]	Large cell carcinoma	3 months	F	49	Fever	Right middle and lower lobectomy, adjuvant chemotherapy	Splenectomy, adjuvant chemotherapy	No evidence of recurrence 7 months after splenectomy
Alloubi (2011)[21]	Adenocarcinoma	12 months	M	58	Abdominal pain	Left upper lobectomy, adjuvant chemotherapy	Splenectomy, adjuvant chemotherapy	Died 8 months after splenectomy
Hepgur (2010)[22]	Adenocarcinoma	0 months	M	74	Abdominal pain	Radiochemotherapy	Best supportive care	Died 10 weeks after initial treatment
Yu (2011)[9]	Adenocarcinoma	0 months	F	56	n.a.	Chemotherapy	Microwave ablation	Multiple metastases at 11 months after ablation. The patient started immunotherapy and is alive 28 months later
Soussan (2011)[51]	Adenocarcinoma	0 months	M	52	n.a.	n.a.	n.a.	n.a.
Dias (2012)[23]	Squamous cell carcinoma	16 months	M	82	Asymptomatic	Right bilobectomy, adjuvant radiochemotherapy	Splenectomy	No evidence of recurrence 12 months after splenectomy
Sardenberg (2013)[24]	Adenocarcinoma	7 months	F	49	Abdominal pain	Right upper lobectomy, adjuvant chemotherapy	Splenectomy, adjuvant chemotherapy	No evidence of recurrence 8 years after splenectomy
Oussama (2013)[52]	Clear cell carcinoma	0 months	M	58	Abdominal pain, deterioration in general health	Palliative chemotherapy	Splenectomy	n.a.
Eisa (2014)[25]	Adenocarcinoma	0 months	F	55	Abdominal pain, fullness sensation	Wedge resection of the lung tumor	Splenectomy, adjuvant chemotherapy	No evidence of recurrence 6 months after splenectomy
Yamane (2015)[26]	Small cell lung cancer	0 months	M	68	n.a.	Radiochemotherapy	Splenectomy	Died 7 months after splenectomy
Belli (2016)[27]	Large cell carcinoma	5 years	M	65	Asymptomatic	Right pneumonectomy	Splenectomy	No evidence of recurrence 16 months after splenectomy
Cai (2015)[28]	Adenocarcinoma	17 months	F	56	Asymptomatic	Neoadjuvant radiochemotherapy, right lower lobectomy	Splenectomy	No evidence of recurrence 1 month after splenectomy
Iguchi (2015)[29]	Adenocarcinoma	12 months	F	63	Asymptomatic	Left lower lobectomy, adjuvant chemotherapy	Splenectomy, adjuvant chemotherapy	No evidence of recurrence at the time of publication
Mitsimponas (2017)[30]	Adenocarcinoma	0 months	F	66	Asymptomatic	Radiochemotherapy	Best supportive care	No evidence of recurrence 13 months after initial treatment
Hara (2017)[31]	Adenocarcinoma	0 months	F	81	Asymptomatic	Partial right upper lobectomy	Splenectomy	No evidence of recurrence 4 years after splenectomy
Nishikawa (2017)[32]	Pulmonary typical carcinoid	7 years	M	73	Asymptomatic	Right upper lobectomy	Splenectomy	No evidence of recurrence 20 months after splenectomy
Zeng (2018)[53]	Adenoid cystic carcinoma	4 years	F	38	Abdominal pain	Right middle lobectomy	Splenectomy	n.a.
Lopera (2018)[54]	Large cell carcinoma	n.a.	F	69	Abdominal pain	Radiochemotherapy	Splenectomy	n.a.
Tanaka (2020)[33]	Squamous cell carcinoma	0 months	M	78	Splenic rupture	Right upper lobectomy, adjuvant chemotherapy	Splenectomy	No evidence of recurrence 15 months after splenectomy
Grant‐Freemantle (2020)[34]	Adenocarcinoma	28 months	F	73	Asymptomatic	Right lower lobectomy	Splenectomy	No evidence of recurrence 9 months after splenectomy
Matsuoka (2021)[35]	Adenocarcinoma	0 months	F	69	Asymptomatic	Right middle lobectomy	Splenectomy	No evidence of recurrence 2 years after splenectomy
Reljic (2022)[36]	Adenosquamous carcinoma	144 months	M	56	Asymptomatic	Left upper lobectomy, adjuvant radiochemotherapy	Splenectomy, adjuvant chemotherapy	No evidence of recurrence 2 years after splenectomy
Xuan (2023)[37]	Squamous cell carcinoma	24 months	M	62	Asymptomatic	Left pneumonectomy, adjuvant chemotherapy	Splenectomy	No evidence of recurrence 3 years after splenectomy
Yu (2024)[38]	Mixed adenocarcinoma and large cell neuroendocrine carcinoma	0 months	M	68	Asymptomatic	Resection of the primary lung tumor	Splenectomy	No evidence of recurrence 1 year after splenectomy
Oshobu (2024)[55]	Small cell neuroendocrine lung cancer	0 months	M	64	n.a.	n.a.	n.a.	n.a.
Beatty (2024)[56]	Non–small cell lung cancer	n.a.	M	76	Asymptomatic	Radiochemotherapy	Splenectomy	The postoperative course was uneventful
Our case	Adenocarcinoma	18 months	F	67	Asymptomatic	Neoadjuvant chemotherapy, right upper lobectomy	Splenectomy	No evidence of recurrence 14 years after splenectomy

The most common histological subtype was adenocarcinoma, reported in 22 cases (44%), followed by squamous cell carcinoma in eight cases (16%) and large cell carcinoma in five cases (10%). Other described subtypes were SCLC, neuroendocrine lung cancer, carcinoid tumors, and mixed carcinomas. Information regarding specific molecular subtypes was rarely provided and remained incomplete. While molecular characterization is now the standard of care in NSCLC diagnostics, the lack of such data in our case and in historical reports represents a significant limitation. It remains to be determined whether specific molecular profiles are preferentially associated with splenic metastasis.

Twenty‐one patients (47%) had synchronous metastases, diagnosed at the time of the primary lung cancer diagnosis or during staging workup, while 24 patients (53%) developed metachronous metastases. On average, metachronous splenic lesions were detected 31 months after the initial lung cancer diagnosis. A notable clustering of metachronous metastases was observed around the 20‐month mark. Additionally, four outliers were observed at 4, 5, 8, and 12 years after the initial lung cancer diagnosis. For five cases, information regarding the timing of metastatic diagnosis was unavailable.

In 24 patients (57%), the splenic metastases were asymptomatic. Most of the splenic metastases were therefore incidentally detected in routine follow‐up scans and staging workup. Among symptomatic cases, the most common complaint was abdominal pain (29%). Other reported symptoms and complications included splenic rupture (four cases, 10%) and fever (one case, 2%). Two cases (5%) were detected upon measurement of an elevated tumor marker. Symptom status was not reported for eight cases.

Treatment primarily consisted of splenectomy, which was performed in 40 patients (91%), either as monotherapy or in combination with chemotherapy (eight cases, 18%) or radiotherapy (two cases, 5%). One patient underwent microwave ablation. Best supportive care was provided in three cases (7%), while treatment details were unavailable for six patients.

**Figure 2:**
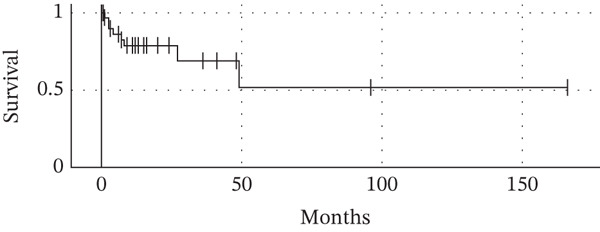
Kaplan–Meier survival curve showing the overall survival for 31 cases of isolated splenic metastasis from lung cancer with published follow‐up data [9–38].

Follow‐up data were available for 31 out of the 50 published case reports. For these cases, we calculated overall survival from the diagnosis of splenic metastasis to death or last follow‐up. A Kaplan–Meier survival curve was generated using the *StatsKingdom* online tool [58] (Figure 2). We acknowledge that survival analyses derived from incomplete retrospective data must be interpreted with significant caution. Nonetheless, considering the typical prognosis for metastatic lung cancer, the outcomes were surprisingly favorable, with several patients achieving long‐term survival spanning multiple years.

Splenic metastasis generally occurs in the context of multivisceral disseminated disease or as an isolated lesion and is considered rare. Data on prevalence are primarily derived from autopsy studies, which report rates ranging from 3% to 7.1% [59–61]. The largest autopsy series, published by Berge [59] in 1974, identified splenic metastases in 312 out of 7165 (7.1%) cancer‐related autopsies. More recently, Milovanovic et al. [5] reported a 4% prevalence of splenic metastases specifically in lung cancer. It is important to interpret these frequencies in their relative context. First, historical data may not reflect today’s clinical reality; both diagnostics and oncological therapies have advanced over the last years, potentially altering the incidence and detection of metastases. Second, autopsy studies are subject to selection bias, focusing on terminal disease stages, which does not reflect general epidemiology. A recent retrospective analysis conducted by Sauer et al. [62] in 2009 found that only 59 out of 29,364 cancer patients (0.96%) had splenic metastases, with solitary metastasis in only three out of those 59 patients. Compérat et al. [63] compiled an overview of all 93 well‐documented case reports of isolated splenic metastases from various primary cancers that were published before 2007. They described the most common entities of isolated splenic metastasis as being colorectal, ovarian, and lung cancer. However, in the broader context of disseminated disease, the main primary sites are the lung, ovary, colon, stomach, and melanoma. Beyond this, literature on isolated splenic metastasis is limited to individual case reports.

Many attempts to explain the rarity of splenic involvement in metastatic cancer have been undertaken over the years. Traditional “mechanical” hypotheses such as high splenic blood flow, the acute angle of the splenic artery, splenic capsular contractions, and the absence of afferent lymphatics hindering metastatic growth have largely lost their relevance. Current oncology suggests that metastases arise from early micrometastatic dissemination, leading to latent metastatic growth [64]. It seems that the splenic microenvironment might play a role in preventing micrometastases from growing into clinically detectable metastases [63]. One theory suggests that cancer cells are destroyed due to exposure to proapoptotic signals in the spleen [7]. This could explain the high prevalence of micrometastases at autopsy in patients with multivisceral disseminated cancer and the rarity of clinically detectable splenic metastases [59].

Consistent with the broader literature stating that over 60% of isolated splenic metastases are asymptomatic [63], our analysis, specifically focusing on lung cancer, revealed that 57% of published cases were clinically silent. The splenic metastasis in our patient was similarly asymptomatic, identified solely via surveillance CT following her primary lung cancer resection. Symptomatic patients usually present with nonspecific abdominal pain. The combination of absent or nonspecific symptoms, such as abdominal pain, which have a broad spectrum of differential diagnoses along with the rarity of splenic metastasis, might lead to splenic lesions being overlooked or misdiagnosed initially. The diagnostic challenge lies in differentiating between the rare case of metastasis and more common primary splenic pathologies such as abscess, hemangioma, or infarction [65]. While ultrasound, CT, MRI, and PET are essential for noninvasive detection and characterization of splenic lesions [66], the clinical history and findings outside of the spleen can help narrow differential diagnoses. Histological examination can be done by fine needle aspiration (FNA), percutaneous core needle biopsy (CNB), and splenectomy [67, 68]. However, an analysis of splenectomies performed for imaging‐detected lesions found that FNA results can be inconclusive. Notably, a history of cancer is the only independent predictor of splenic malignancy [69]. Consequently, patients with a history of cancer and a high probability of splenic metastasis may especially benefit from splenectomy since it can provide both diagnostic and potential therapeutic benefits.

Given the absence of clinical guidelines for the situation of isolated splenic lesions in patients with a history of lung cancer, this case report suggests that isolated splenic metastasis can be diagnosed and treated at the same time through splenectomy in well‐selected patients. Obviously, a surgical intervention such as splenectomy is generally not indicated in patients with multivisceral disseminated end‐stage malignant disease because of the poor overall prognosis. However, splenectomy may be a viable option in selected cases of isolated splenic metastasis and symptomatic metastasis for the purpose of symptom relief [69].

For lung cancer with isolated splenic metastasis, the limited number of reported cases with adequate follow‐up precludes a definitive statement regarding the general survival benefit of splenectomy. Nevertheless, our case demonstrates an example of an exceptional outcome, with a long‐term survival of 14 years following splenectomy. It is highly improbable that our patient would have achieved this without the surgical resection of the splenic metastasis. For smaller metastases, microwave ablation might be a viable locally targeted alternative in specialized centers [9].

Splenectomy and the asplenic state carry risks of infectious and noninfectious complications. The risk of postsplenectomy infection is approximately 7% [70, 71]. The most feared long‐term risk is overwhelming postsplenectomy infection (OPSI), which, although relatively rare, has a high mortality rate of up to 50% or higher. The risk of severe infection and splenectomy‐related death is highest within the first 3 years postsplenectomy [72]. The likelihood of certain complications is influenced by multiple factors such as patient age, general health status, and the indication for splenectomy (e.g., trauma, oncological, and hematological). Consequently, preventive measures for infectious complications include antibiotic prophylaxis, vaccinations, and patient education and are indicated pre‐ and postsplenectomy [73].

Our patient remained free of major infectious complications for many years postsplenectomy, supporting the feasibility of our treatment approach in well‐selected cases. At age 81, 14 years after her splenectomy, she died from causes related to left‐sided heart failure and infection. Whether her asplenic state contributed to the severity of her final infection remains speculative.

We acknowledge the limitations of this review, including its retrospective design, the small sample size, and the incomplete data inherent in published case reports.

## 4. Conclusion

Isolated splenic metastasis from lung cancer is exceedingly rare and poses a great diagnostic challenge. The remarkable 14‐year survival of our patient represents, to our knowledge, the longest documented survival for isolated splenic lung cancer metastasis. While no clinical guidelines for the management of this metastatic pattern exist, we believe that our case supports considering splenectomy as both a diagnostic and therapeutic intervention in well‐selected cases and that it may lead to relevant long‐term survival.

## Funding

No funding was received for this manuscript.

## Ethics Statement

The authors have nothing to report.

## Consent

Written informed consent for the publication of the medical case details and any accompanying images was obtained from the patient.

## Conflicts of Interest

The authors declare no conflicts of interest.

## Data Availability

Data are available upon request.
